# Supplementary material for: Cervical Screening in North Sardinia (Italy): Genotype Distribution and Prevalence of HPV among Women with ASC-US Cytology
Source: Int J Environ Res Public Health. 2022 Jan 8;19(2):693. doi: 10.3390/ijerph19020693 (PMC8775344; doi:10.3390/ijerph19020693)
Supplement: Supplementary file 1 [file ijerph-19-00693-s001.zip › ijerph-1501221-supplementary.pdf]

International Journal of
Environmental Research and Public Health

MDPI

Table S1: Distribution of HPV genotypes by age groups

	Age groups at baseline								
	**25-29 years (n= 215)**	**30-34 years (n= 185)**	**35-39 years (n= 160)**	**40-44 years (n= 202)**	**45-49 years (n= 188)**	**50-54 years (n= 115)**	**55-59 years (n= 65)**	**>60 years (n= 56)**	**p-value**
**Hpv-16,** n (%)	32 (14.9)	28 (15.1)	27 (16.9)	26 (12.9)	38 (20.2)	34 (29.7)	11 (16.9)	11 (19.6)	0.01
**Hpv-18,** n (%)	12 (5.6)	16 (8.7)	8 (5.0)	9 (4.5)	4 (2.1)	5 (4.4)	4 (6.2)	2 (3.6)	0.25
**Hpv-31,** n (%)	18 (8.4)	17(9.2)	18 (11.3)	12 (5.9)	17 (9.0)	11 (9.6)	3 (4.6)	11 (19.6)	0.08
**Hpv-33,** n (%)	4(1.9)	2 (1.1)	1 (0.6)	4 (2.0)	2 (1.1)	2 (1.7)	0 (0.0)	1 (1.8)	0.91
**Hpv-35,** n (%)	4 (1.9)	3 (1.6)	3 (1.9)	3(1.5)	1 (0.5)	1 (0.9)	3 (4.6)	3 (5.4)	0.22
**Hpv-39,** n (%)	12 (5.6)	11 (6.0)	10 (6.3)	9 (4.5)	7 (3.7)	5 (4.4)	4 (6.2)	1 (1.8)	0.87
**Hpv-45,** n (%)	4(1.9)	1 (0.5)	2 (1.3)	1 (0.5)	0 (0.0)	3 (2.6)	1 (1.5)	2 (3.6)	0.11
**Hpv-51,** n (%)	10 (4.7)	13 (7.0)	9 (5.6)	10 (5.0)	10 (5.3)	5 (4.4)	2 (3.1)	2 (3.6)	0.96
**Hpv-52,** n (%)	9 (4.2)	8 (4.3)	9 (5.6)	6 (3.0)	7 (3.7)	9 (7.8)	4(6.2)	3 (5.4)	0.60
**Hpv-56,** n (%)	15 (7.0)	9 (4.9)	12 (7.5)	8 (4.0)	8 (4.3)	10 (8.7)	2 (3.1)	3 (5.4)	051
**Hpv-58,** n (%)	10 (4.7)	6 (3.2)	5 (3.1)	9 (4.5)	5 (2.7)	4 (3.5)	2 (3.1)	2 (3.6)	0.98
**Hpv-59,** n (%)	9 (4.2)	4 (2.2)	5 (3.1)	7 (3.5)	2 (1.1)	4 (3.5)	4 (6.2)	3 (5.4)	0.33
**Hpv-66,** n (%)	15 (7.0)	8 (4.3)	6 (3.8)	14 (6.9)	8(4.3)	8 (7.0)	6 (9.2)	3 (5.4)	0.57
**Hpv-68,** n (%)	9 (4.2)	9 (4.9)	6 (3.8)	10 (5.0)	9 (4.8)	5 (4.4)	6 (9.2)	1 (1.8)	0.77
**Positivity for at least one HR-HPV genotype,** n (%)	104 (48.4)	90 (48.7)	83 (51.9)	84 (41.6)	83 (44.2)	68 (59.1)	28 (43.1)	31 (55.4)	0.07

HPV-positivity <41 y.o, n (%)	*320/537 (59.6)*	*p-value <0.001*
HPV-positivity ≥41 y.o, n (%)	*251/649 (38.7*	

Table S2. Positivity distribution by age groups and civil-status

25-34 age-group	**Not Vaccinated (n=277)**	**Vaccinated (n=123)**	**p-value**
**Hpv-16, n (%)**	37 (13.4)	23 (18.7)	0.17
**Positivity for at least one HR-HPV genotype, n (%)**	94 (33.9)	100 (81.3)	<0.001

35-44 age-group	**Not Vaccinated (n=272)**	**Vaccinated * (n=90)**	**p-value**
**Hpv-16, n (%)**	36 (13.2)	17 (18.9)	0.19
**Positivity for at least one HR-HPV genotype, n (%)**	90 (33.1)	77 (85.6)	<0.001

45-54 age-group	**Not Vaccinated (n=235)**	**Vaccinated * (n=68)**	**p-value**
**Hpv-16, n (%)**	59 (25.1)	13 (19.1)	0.31
**Positivity for at least one HR-HPV genotype, n (%)**	101 (43.0)	50 (73.5)	<0.001

≥55 age-group	**Not Vaccinated (n=91)**	**Vaccinated * (n=30)**	**p-value**
**Hpv-16, n (%)**	18 (19.8)	4 (13.3)	0.43
**Positivity for at least one HR-HPV genotype, n (%)**	36 (39.6)	23 (76.7)	<0.001

Single	**Not Vaccinated (n=314)**	**Vaccinated * (n=182)**	**p-value**
**Hpv-16, n (%)**	59 (18.8)	33 (18.1)	0.86
**Positivity for at least one HR-HPV genotype, n (%)**	143 (45.5)	151 (83.0)	<0.001

Married	**Not Vaccinated (n=269)**	**Vaccinated * (n=55)**	**p-value**
**Hpv-16, n (%)**	34 (12.6)	8 (14.6)	0.71
**Positivity for at least one HR-HPV genotype, n (%)**	65 (24.2)	37 (67.3)	<0.001

General results	**Not Vaccinated (n=875)**	**Vaccinated * (n=311)**	**p-value**
**Hpv-16, n (%)**	150 (17.1)	57 (18.3)	0.64

**Positivity for at least one HR-HPV genotype, n (%)**	321 (36.7)	250 (80.4)	<0.001
